# Sub-acute Toxicity Assessment of Taxol Isolated From *Fusarium Solani*, an Endophytic Fungus of *Taxus Brevifolia*, in Wistar Rats and Analyzing Its Cytotoxicity and Apoptotic Potential in Lung Cancer Cells

**DOI:** 10.3389/fonc.2020.538865

**Published:** 2020-10-06

**Authors:** Gini C. Kuriakose, B. P. Arathi, Mangalath Divya Lakshmanan, M. V. Jiby, Ramachandra Subbaraya Gudde, C. Jayabhaskaran

**Affiliations:** ^1^Department of Biochemistry, Indian Institute of Science, Bangalore, India; ^2^Central Animal Facility, Indian Institute of Science, Bangalore, India

**Keywords:** taxol, sub-acute toxicity, cell cycle arrest, mitochondria membrane potential loss, apoptosis, *F. solani*

## Abstract

The limited availability of taxol from plant sources has prompted the scientific world to look for an alternative, as in the chemical synthesis of tissue cultures of the *Taxus* species, to meet the increasing demand for the drug. However, these alternative means are expensive or result in low yield. Previously, we have reported that *Fusarium solani* isolated from *Taxus celebica* produced taxol and its precursor baccatin III in liquid-grown cultures, and it exhibited promising anticancerous effects in certain cancer cell lines. In the present study, we examined the sub-acute toxicity of fungal taxol (FS) in Wistar rats according to the Organization for Economic Co-operation and Development (OECD) guidelines. The sub-acute oral administration of FS up to 500 mg/kg for a period of 28 days appears to be safe in rats and did not cause severe treatment-related toxicity or treatment-related death. The observed changes in body weight, histopathology, hematological and biochemical parameters, and organ weight were not significant compared to those in the control group of animals. The results suggest that FS is relatively safe when administered orally in rats. The antiproliferative and apoptosis-inducing activities were studied in A549 (human lung cancer) cell line. FS arrested the cells at S and G2/M phases, leading to apoptosis. The characteristic molecular signatures of apoptosis, such as externalized phosphatidyl serine, DNA fragmentation, and nuclear and chromatin condensation, were observed upon FS treatment. FS triggered the generation of reactive oxygen species in A549 cells and elicited cell death by both extrinsic as well as the mitochondria-mediated intrinsic pathway of apoptosis. These results indicate that endophytic fungi isolated from medicinal plants may serve as potential sources of anticancerous compounds with little side effects.

## Introduction

Taxol is a diterpenoid isolated initially from the stem or bark of the Pacific yew tree (*Taxus brevifolia*). Taxol is used as an anticancer drug for several different types of cancer such as breast cancer, ovarian cancer, prostate cancer, non-small cell lung cancers, adenocarcinoma, and esophagus squamous cell carcinoma (1–6). Low yields of taxol from the plants encouraged researchers to look for alternate ways of taxol production, which led to the identification of endophytic fungi as sources of taxol (7). Due to an increase in demand, it is essential to isolate taxol from endophytic fungi or by microbe fermentation method to obtain a large quantity. Many studies have focused on the isolation of endophytic fungi from medicinal plants, discovering many new endophytic fungal species such as *Taxomyces andreanae, Taxodium disticum, Tubercularia* sp., *Pestalotiopsis microspora, Alternaria sp*., *Fusarium maire*, and *Periconia* sp., some of which have potential to be used in the production of medicines (8–12). Previously, we have demonstrated the effect of taxol from *Fusarium solani*, an endophytic fungus of *Taxus brevifolia*, on cancer cell inhibition and apoptosis induction in various human carcinoma cell lines (13). We also reported the apoptotic mechanisms of fungal taxol and its precursor baccatin III in cancer cells (13). Taxol attains its antitumor activity by promoting tubulin dimerization and inhibiting the depolymerization of microtubules, resulting in the formation of abnormally stable and non-functional microtubules (14, 15). With this action, exposure to taxol prevents the completion of mitosis, resulting in mitotic metaphase arrest and cellular toxicity (16). Despite these reports, the precise mechanism of taxol-induced apoptosis remains poorly understood.

In the present study, we investigated the apoptosis-inducing activity of taxol isolated from the fungus *F. solani in* A549 cancer cell line, and its toxicological studies through oral route were carried out in animal models. Lung cancer is a leading cause of cancer-related deaths, resulting in more than one million deaths globally every year. It is greater than the death rates attributed by colorectal, breast, and prostate cancers combined.

Oral plaxitaxel has entered phase III clinical trial and is found effective (17–19). Sub-acute toxicity studies should, however, be carried out before clinical trial, and it has been previously reported for many natural extracts and products (20–22). Experimental data on the toxicity profile of taxol from endophytic fungi should be obtained to increase assurance on their safety and on the development of pharmaceuticals (23). However, oral paclitaxel has low bioavailability because it is a substrate of the intestinal P-gp pump. Tween 80 is a noteworthy *P*-*gp* efflux *pump* inhibitor (24) that increases the absorption of oral paclitaxel. Here, we have evaluated the sub-acute toxic effects of fungal taxol administered through oral route with Tween 80 at 2% as vehicle in an animal model and elucidated the molecular mechanism of FS-induced apoptosis in non-small cell lung cancer (NSCLC) cell line A549.

## Materials and Methods

### Extraction of Taxol From Endophytic Fungi *F. solani* Isolated From *Taxus celebica*

Taxol, purified from *F*. *solani*, isolated from the stem cutting of *T. brevifolia* (25) previously from our laboratory was used in the study. The fungi were identified by morphological as well as internal transcribed spacer (ITS) and D1/D2 26S rDNA sequence analysis (25). Taxol was identified based on high-performance liquid chromatography (HPLC) by comparing the retention time to standard peaks (25). The purified taxol, referred to as FS (*F. solani* taxol) was used for sub-acute toxicity studies and further investigation on A549, a lung NSCLC cell line.

### Animal Ethical Clearance Statement

All investigations were performed at the central animal facility after approval of the institutional animal ethics committee of the Indian Institute of Science, Bangalore, India.

### Animal Housing and Maintenance

Adult male and female Wistar rats (10–12 weeks, weighing 180–200 g) from the Central Animal Facility, Indian Institute of Science, were used for the study. They were housed under controlled temperature (23–25°C), with a constant 12-h light–dark cycle and free access to food and water. A total of 40 animals (females and males) were used for the sub-acute toxicity test (26, 27).

### Sub-acute Toxicity Studies of FS

The animals were divided into four experimental groups (*n* = 10 animals/group, five males and five females). Two different doses of FS (125 and 250 mg/kg) were administered per group orally, by using an oral gauge, daily for 28 consecutive days. The control group received only the vehicle (saline with Tween® 80 at 2%). Another group (satellite group) received the maximum dose of 500 mg/kg of FS for 28 days and remained untreated for 14 more days. It is important to use a satellite group for observation of reversibility, persistence, or delayed occurrence of toxic effects related to the administration of the test substance. The doses were chosen based on Guideline 407 from OECD (repeated dose 28-day oral toxicity study in rodents) (27).

### Investigation of Hematology and Biochemical Parameters

For the hematological investigation, all animals were fasted overnight but were allowed access to water. The rats were anesthetized, and blood samples were drawn from the abdominal aorta. Whole blood was collected in a tube containing ethylenediaminetetraacetic acid (EDTA) and processed immediately for hematological analysis. The parameters included red blood cell count, hematocrit, hemoglobin, leucocytes, erythrocytes, and platelet count. The hematological analysis was performed using an automatic hematological analyzer (Sysmex KX-21). For the measurement of biochemical parameters, blood samples were collected in tubes without EDTA, kept at 4°C for 4 h to let it clot, and centrifuged at 3,000 rpm at 5°C for 15 min to separate the serum. The serum samples were used for evaluating the different biochemistry parameters such as total serum protein, alanine aminotransferase, aspartate aminotransferase, urea, creatinine, and total cholesterol using standard Erba diagnostic kits using Erba Mannheim, Chem-7 analyzer, Mennheim, Germany.

### Histopathological Investigation

The tissues of liver, lung, and kidney obtained from both control and experimental groups were fixed in 10% buffered formalin (pH 7.4). The tissues were then dehydrated in a graded series of ethanol (70–100%) and finally embedded in paraffin wax. Later, 5-μm-thin sections were prepared using a microtome (Leica RM2235) and stained with hematoxylin and eosin for microscopic examination. Histological sections were examined using a standard light microscope, and photomicrographs were taken.

Organs of the sacrificed animals, namely, heart, liver, kidneys, lung, spleen, testes, and uterus, were removed surgically, cleaned with ice-cold saline solution, placed on absorbent papers, and then weighed to obtain the absolute organ weight. The relative organ weight of each animal was then calculated using the formula: relative organ weight = absolute organ weight ÷ body weight at sacrifice × 100%.

### Cell Culture

The A549 cells (lung carcinoma cell line) and the HEK-293 cells (normal human embryonic kidney cells) used for the experiments were procured from the National Center for Cell Sciences (NCCS), Pune, India. The cells were cultured in Dulbecco's modified Eagles medium supplemented with 10% fetal bovine serum. The cell line was maintained in a humidified atmosphere of 5% CO_2_ and 95% air at 37°C. A total of 1 × 10^5^ cells were cultured in 1 ml of the same medium in 24-well-plates at 37°C in a humidified 5%-CO_2_ incubator for 24 h prior to the experiments.

### Determination of Cell Viability, Cell Cycle Distribution, and Apoptosis in FS-Treated A549 Cells

A549 and HEK-293 cells were treated with FS at the indicated concentration and time in each experiment. Cell viability was determined by MTT assay as described previously (28). Cell cycle analysis was done using propidium iodide (PI) staining at an early time point of 6 h as previously described (28). Detection of the morphological changes associated with apoptosis was assessed by 4′,6-diamidino-2-phenylindole (DAPI) staining. The production of reactive oxygen species (ROS) was measured by flow cytometry using the dye 2′,7′-dichlorodihydrofluorescein diacetate (DCFH-DA) as described previously (28). To quantify the percentage of cells undergoing apoptosis, we used annexin V–fluorescein isothiocyanate (FITC) and PI at a late time point of 24 h as described previously (28).

### Western Blot Analysis

A549 cells were grown in a monolayer in a 60-mm dish (3 × 10^6^ cells) followed by treatment with appropriate concentrations of FS and then processed for protein extraction. For extraction, the floating cells and the adherent cells were collected and pelleted by centrifugation. The cells were lysed by radioimmunoprecipitation assay buffer [50 mM tris-HCl, pH 7.4, 150 mM NaCl, 1 mM EDTA, 1 mM phenylmethanesulfonyluoride, 1 mM sodium orthovanadate, 1 mM sodium fluoride, 1% Nonidet P-40 (NP-40), 0.25% deoxycholate, 0.1% sodium dodecyl sulfate (SDS), 1 × protease inhibitor cocktail] and then centrifuged (10,000 rpm) for 10 min at 4°C to obtain the supernatant. The total protein content was determined by Bradford method. Thirty micrograms of protein samples was separated on 10% SDS polyacrylamide gel and then transferred electrophoretically to nitrocellulose membrane. The membrane was treated for 1 h with a blocking solution and incubated overnight at 4°C with primary antibodies [β-actin, Bax, Bcl-2, p53, and apoptotic peptidase activating factor 1 (Apaf-1)]. Subsequently, blots were probed with the secondary antibodies horseradish peroxidase-conjugated goat antirabbit/mouse immunoglobulin G. The membranes were again washed thrice (5 min each) with phosphate-buffered saline with Tween®. Freshly prepared enhanced chemiluminescence solution (ECL Plus kit, PerkinElmer, USA) was added onto the membrane, and the immune-reactive bands were imaged using an Image Quant LAS 4000 digital imaging system (Fuji Film, USA).

### Caspase Activity Assays

The activities of caspase-3, caspase-8, and caspase-9 were determined using corresponding fluorogenic substrates. The cells were treated with different concentrations of FS for 24 h and lysed with cell lysis buffer. The protein concentrations in the cell lysates were determined using the Bradford protein estimation kit (Bio-Rad Laboratories). Two hundred micrograms of cell lysates was added to the reaction buffer with the appropriate caspase fluorogenic substrate (Ac-DEVD-MCA for caspase-3, Ac-IETD-MCA for caspase 8, and Ac-LEHD-MCA for caspase-9) and incubated for 2 h at 37°C. Liberation of MCA was determined using the Tecan pro 200 fluorescence microplate reader that allowed for light excitation at 400-nm wavelength and collected emitted light at a wavelength of 505 nm. The untreated cells were used as a control.

### Statistical Analysis

Data are expressed as the means ± standard deviation of the obtained results. Statistical analysis was performed by one-way analysis of variance with Dunnett's *post hoc* test to evaluate significant differences between groups. Graph Pad Prism version 6.0 for Windows was used for statistical analysis. Data analyses from male and female groups were done separately, and the differences were considered as statistically significant at *p* < 0.05.

## Results and Discussion

Taxol, purified from *F. solani* and isolated from the stem cutting of *T. brevifolia* (25) previously from our laboratory, was used in the study. The fungus was identified by morphological as well as ITS and D1/D2 26S rDNA sequence analysis (25). Taxol was identified based on HPLC by comparing the retention time to the standard peaks (25). Previously we compared the efficiency of FS with the standard commercially available taxol and found that FS has more apoptotic-inducing ability (13, 25). This work was carried out to understand the sub-acute toxicity of FS and the molecular mechanism of FS-induced apoptosis in A549 cells.

### Food, Water Consumption, Body Weight, and General Physic of Wistar Rats Administered With a Sub-acute Dose of FS Remained Stable in Both Male and Female

No treatment-related deaths were observed at any dose up to the maximum of 500 mg/kg during the repeated oral treatment for 28 days. Observations on the general physic of the animals during the treatment phase suggest no apparent alteration in posture, gait, and response to handling. In addition, no changes in skin, fur color, eyes, and mucous membrane were observed with the treatment. There were no observable changes in secretions or excretions in the tested animals.

The food consumption and the water intake of the rats treated with taxol were recorded and compared with the control ones. We observed no statistical alteration in food consumption or water intake (Table 1), illustrating that the FS has no obvious effect on food consumption. The body weights of the control and the FS-treated groups are presented in Table 1. No statistically significant difference in body weight was noted between the control and the treated groups for female and male rats throughout the study period. Moreover, no toxicity signs or animal deaths were observed at any dose up to the maximum of 500 mg/kg during the repeated oral treatment for 28 days.

**Table 1:** Effect of fungal taxol on body weight changes and food and water intake in control and treated Wistar rats in the sub-acute toxicity study.

**Sub-acute toxicity**				
	**Control**	**125 mg**	**250 mg**	**500 mg/kg**
**Female**				
Initial weight (g)	208.21 ± 22.34	200.16 ± 12.54	201.23 ± 16.45	202.60 ± 12.65
Final weight (g)	252.04 ± 19.45	242.19 ± 28.56	229.35 ± 22.24	232.36 ± 25.98
**Body weight gain (%)**				
Food intake (g/day)	129.03 ± 14.56	101.24 ± 12.32	108.59 ± 15.07	102.63 ± 10.54
Water intake (ml/day)	220.10 ± 23.87	230.48 ± 22.56	228.33 ± 14.79	219.32 ± 15.45
**Male**				
Initial weight (g)	272.03 ± 17.45	260.46 ± 18.35	254.55 ± 27.01	259.36 ± 22.02
Final weight (g)	389.22 ± 34.65	394.10 ± 30.56	382.35 ± 25.55	390.09 ± 26.65
**Body weight gain (%)**				
Food intake (g/day)	145.04 ± 12.67	144.28 ± 10.43	156.34 ± 24.21	146.78 ± 10.86
Water intake (ml/day)	252.16 ± 14.34	246.71 ± 15.57	240.46 ± 22.08	256.75 ± 23.75

*Values are expressed as mean ± SEM for 10 animals per group*.

### Hematological and Biochemical Parameters in Wistar Rats Remained Normal With a Sub-acute Dose of FS

The effect of sub-acute oral administration of FS showed no significant (*p* < 0.05) difference in the hematological and biochemical parameters compared to those of the control group (Tables 2, 3). No significant differences among all groups were noted in the level of total bilirubin, total protein, and albumin. Furthermore, there were no significant variations observed in CRA and urea of the FS-treated group of rats when compared to the control group.

**Table 2:** Hematological parameters of female and male Wistar rats treated orally with fungal taxol in the sub-acute toxicity study.

**Sub-acute toxicity**				
	**Control**	**125 mg**	**250 mg**	**500 mg/kg**
**Female**				
Leukocytes (10^3^/μl)	3.52 ± 1.08	3.16 ± 0.67	3.71 ± 0.45	3.79 ± 0.55
Erythrocytes (10^6^/μl)	7.61 ± 0.98	7.93 ± 0.89	7.99 ± 0.78	8.02 ± 0.69
Hemoglobin (g/dl)	14.36 ± 1.59	13.28 ± 2.56	14.44 ± 1.78	13.92 ± 4.78
Hematocrit (%)	45.34 ± 7.35	46.72 ± 6.87	42.33 ± 4.44	44.56 ± 3.98
Platelets (10^3^/μl)	642.33 ± 98.97	640.78 ± 77.09	616.96 ± 97.63	624.78 ± 100.54
**Male**				
Leukocytes (10^3^/μl)	4.02 ± 0.07	4.01 ± 1.45	4.26 ± 1.09	4.11 ± 0.87
Erythrocytes (10^6^/μl)	8.41 ± 1.45	8.23 ± 0.99	8.16 ± 0.54	8.28 ± 1.32
Hemoglobin (g/dl)	14.71 ± 2.56	13.15 ± 1.45	13.24 ± 2.67	13.39 ± 0.99
Hematocrit (%)	46.52 ± 4.56	47.08 ± 5.67	47.24 ± 4.67	46.31 ± 1.56
Platelets (10^3^/μl)	624.03 ± 102.45	619.11 ± 77.98	618.24 ± 89.45	620.69 ± 90.64

*Values are expressed as mean ± SEM for 10 animals per group*.

**Table 3:** Effect of fungal taxol on the biochemical parameters of the control and the treated female and male Wistar rats in the sub-acute toxicity study.

**Sub-acute toxicity**				
	**Control**	**125 mg**	**250 mg**	**500 mg/kg**
**Female**				
Aspartate aminotransferase (U/L)	78.33 ± 4.56	67.39 ± 5.51	74.13 ± 8.45	77.68 ± 6.78
Alanine aminotransferase (U/L)	41.76 ± 9.56	43.22 ± 7.86	48.52 ± 6.88	42.38 ± 8.76
Direct protein (mg/dl)	0.04 ± 0.003	0.03 ± 0.005	0.02 ± 0.001	0.02 ± 0.004
Blood urea nitrogen (mg/dl)	61.94 ± 4.55	53.25 ± 2.99	59.29 ± 6.34	58.78 ± 7.46
Creatinine (mg/dl)	0.21 ± 0.05	0.23 ± 0.002	0.25 ± 0.005	0.20 ± 0.001
Cholesterol (mg/dl)	65.32 ± 3.99	69.26 ± 6.87	71.35 ± 4.76	63.25 ± 4.56
Total protein (g/dl)	7.32 ± 1.76	7.49 ± 2.08	7.58 ± 0.87	7.09 ± 0.95
**Male**				
Aspartate aminotransferase (U/L)	82.66 ± 11.87	86.52 ± 9.56	85.09 ± 12.76	80.36 ± 10.76
Alanine aminotransferase (U/L)	45.67 ± 4.54	40.76 ± 6.87	47.35 ± 3.76	44.96 ± 5.75
Direct protein (mg/dl)	0.05 ± 0.006	0.04 ± 0.001	0.03 ± 0.002	0.04 ± 0.006
Blood urea nitrogen (mg/dl)	55.36 ± 3.56	53.24 ± 6.78	55.76 ± 4.44	52.04 ± 4.76
Creatinine (mg/dl)	0.28 ± 0.07	0.29 ± 0.04	0.34 ± 0.007	0.25 ± 0.001
Cholesterol (mg/dl)	75.46 ± 8.67	73.35 ± 5.87	70.64 ± 6.87	77.96 ± 9.56

*Values are expressed as mean ± SEM for 10 animals per group*.

### Relative Organ Weight of Treated Wistar Rats Assessed for Sub-acute Toxicity Exhibited No Significant Difference Compared to Control

Data related to the absolute and the relative organ weights for both male and female rats treated with FS are presented in Tables 1, 2. A gross examination of internal organs of the control and the treated groups, including, heart, spleen, liver, lung, spleen, kidneys, testis (males), and uterus (females), did not reveal any abnormal findings related to the administration of FS. The absolute and the relative organ weights of the FS treatment group and the control group did not show a significant difference in either sex (Table 4).

**Table 4:** Effect of fungal taxol on the relative organ weight of control and treated Wistar rats in the sub-acute toxicity study.

**Sub-acute toxicity**				
	**Control**	**125 mg**	**250 mg**	**500 mg/kg**
**Female**				
Liver	3.94 ± 0.67	3.75 ± 0.77	3.88 ± 0.65	3.59 ± 0.54
Kidney	0.58 ± 0.11	0.52 ± 0.03	0.49 ± 0.06	0.42 ± 0.09
Spleen	0.23 ± 0.07	0.21 ± 0.09	0.26 ± 0.05	0.21 ± 0.003
Heart	0.36 ± 0.04	0.33 ± 0.05	0.36 ± 0.007	0.34 ± 0.005
Lung	0.62 ± 0.10	0.60 ± 0.01	0.59 ± 0.08	0.63 ± 0.08
Uterus	0.17 ± 0.02	0.15 ± 0.02	0.18 ± 0.002	0.14 ± 0.003
**Male**				
Liver	4.08 ± 0.78	4.12 ± 0.98	4.09 ± 0.34	4.16 ± 0.87
Kidney	0.62 ± 0.008	0.59 ± 0.07	0.61 ± 0.04	0.66 ± 0.005
Spleen	0.25 ± 0.008	0.28 ± 0.02	0.28 ± 0.002	0.31 ± 0.05
Heart	0.39 ± 0.05	0.36 ± 0.005	0.38 ± 0.04	0.39 ± 0.08
Lungs	0.64 ± 0.06	0.61 ± 0.04	0.63 ± 0.007	0.65 ± 0.009
Testis	0.41 ± 0.006	0.42 ± 0.003	0.48 ± 0.009	0.46 ± 0.06

*Values are expressed as mean ± SEM for 10 animals per group*.

### Histopathology Analysis of FS-Treated Group Exhibited No Significant Difference in Morphologic Characteristics

**Figure 1:**
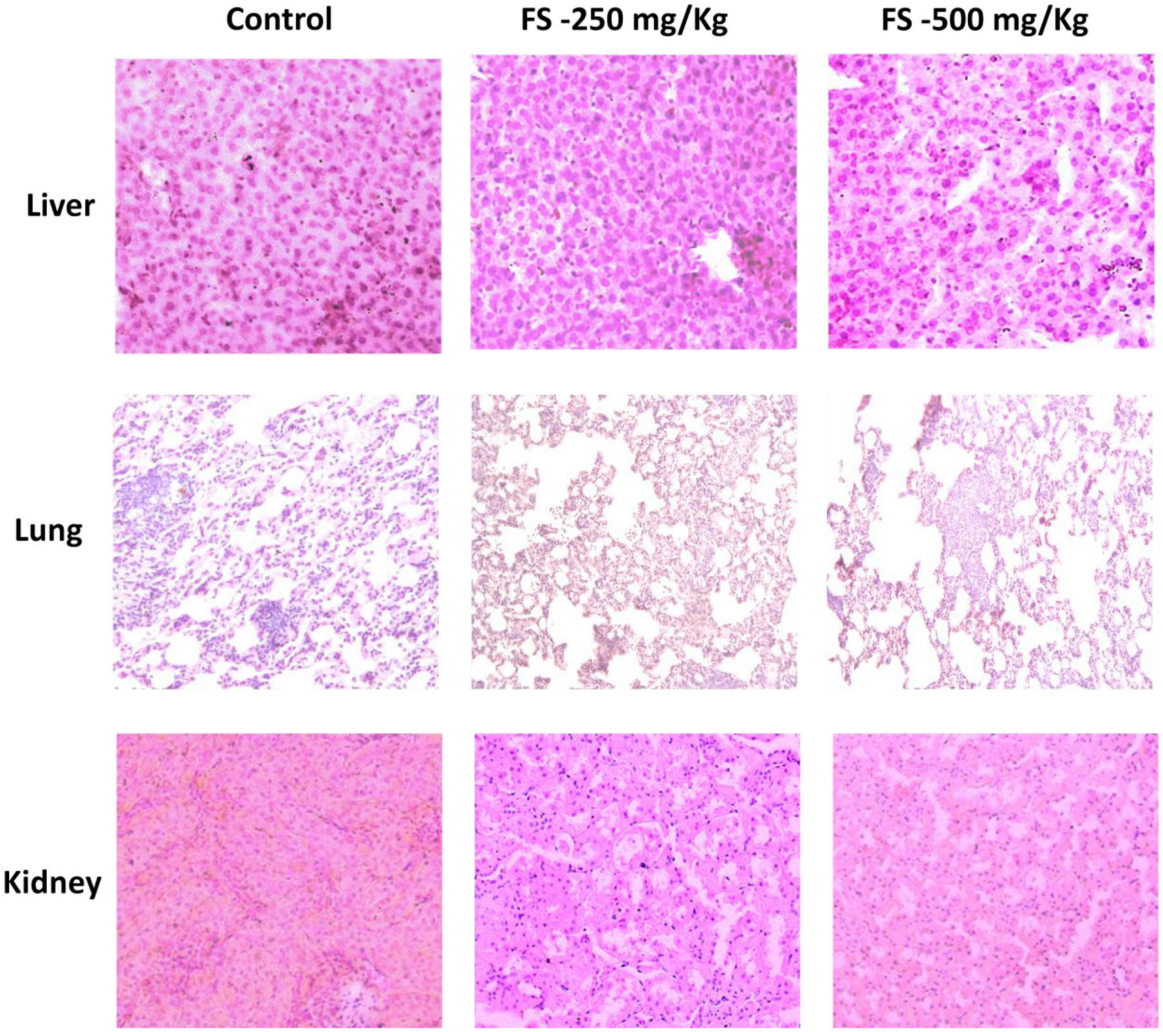
Hematoxylin–eosin-stained photomicrographs of liver, lung, and kidney tissues of Wistar rats that were administered with 250 or 500 mg/kg of fungal taxol during the sub-acute toxicity study.

At the end of treatment, vital organs such as liver, lungs, and kidney were subjected to histopathological examination. The microscopic observation showed slight treatment-related alterations as pathological changes for kidney and lung tissues in the FS-treated group compared to the control group (Figure 1) at a very high concentration of 500 mg/kg. Except for slight granular infiltration, the liver appeared almost normal in the treated group compared to the control. Mild tubular damage of the kidney is observed in the treated group. However, this did not affect the activity nor induced death in the satellite group. Other biochemical and hematological parameters were found to be normal, which suggest that FS may be safe for treatment. The sub-acute oral administration of FS up to 500 mg/kg may not cause severe treatment-related toxicity. Following the sub-acute toxicity studies, we analyzed in detail the apoptosis potential of FS in lung NSCLC cell line.

### FS Reduces Cell Proliferation by Arresting Cells at S and G2/M Phases of the Cell Cycle, Leading to Apoptosis

**Figure 2:**
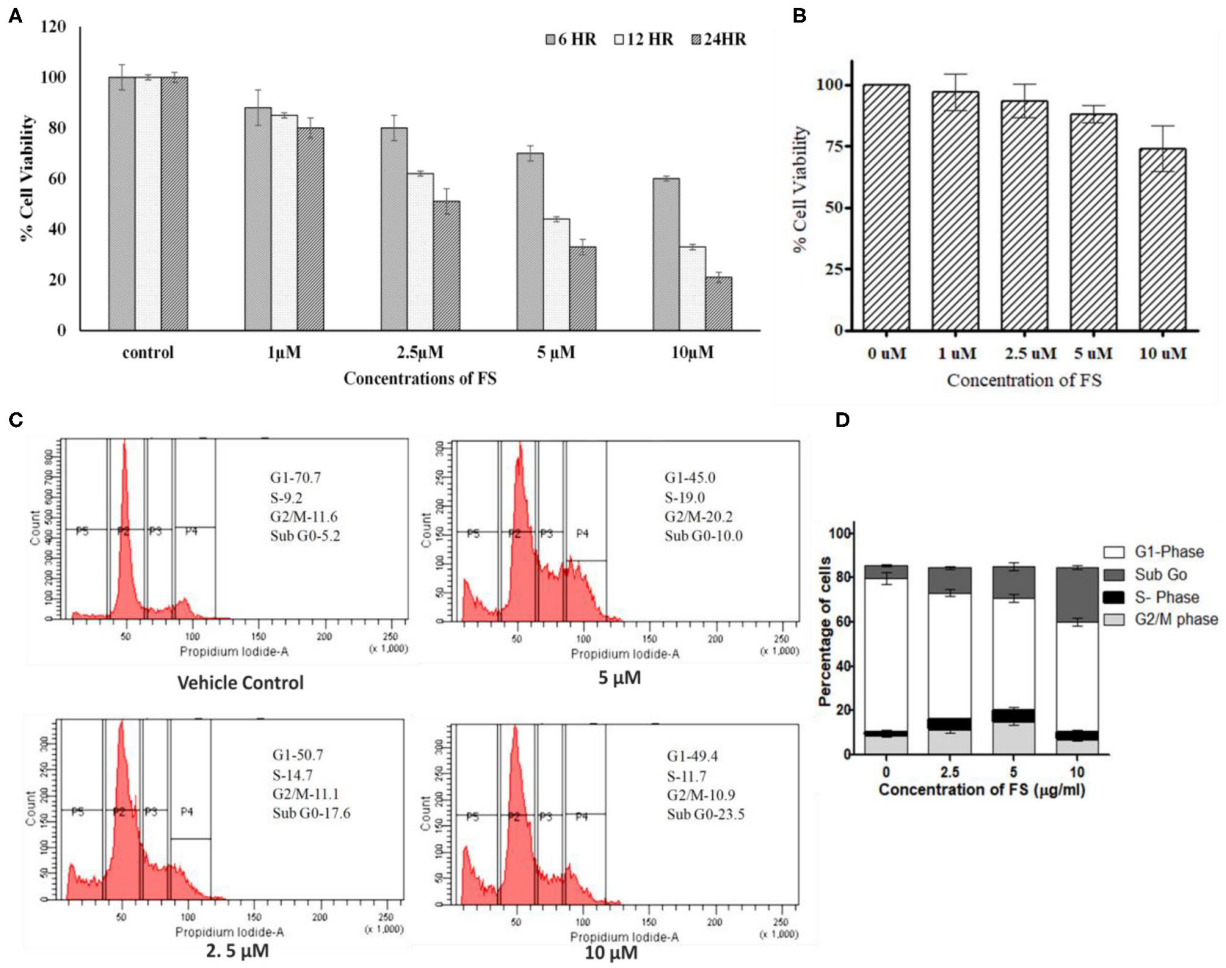
Effect of fungal taxol (FS) on the growth and cell cycle distribution in A549 cells. **(A)** Effect of FS on cell proliferation. Lung cancer cell line A549 cells were treated with different concentrations (5, 10, and 20 μM) of FS for 24 h. Cell viability was determined by MTT assay at 6, 12, and 24 h and expressed as percent of the control value. Dimethyl sulfoxide (DMSO)-treated (0.1%) cells served as vehicle control. Data are the means ± SD of three independent experiments. **(B)** Normal HEK-293 cells treated with different concentrations (5, 10, and 20 μM) of FS for 24 h remain unaffected. Cell viability was determined by MTT assay at 24 h and expressed as percent of the control value. DMSO-treated (0.1%) cells served as vehicle control. Data are the means ± SD of three independent experiments. **(C)** Effect of FS on cell cycle progression on A549 cells at 6 h. The cells were treated with different concentrations of FS (2.5, 5, and 10 μM) for 24 h and subsequently analyzed by flow cytometry using propidium iodide staining to determine the cell cycle. The cell cycle distributions at sub-G0, G1, S, and G2/M phases are represented. The subG0 phase represents cells undergoing apoptosis or cell death. **(D)** The bar diagram represents the population of cells at different phases of the cell cycle. Data are the means ± SD of three independent experiments.

An anti-proliferative assay by MTT carried out for FS indicated a significant inhibition in the proliferation of A549 cells in a dose- and time-dependent manner. As shown in Figure 2A, we observed a significant reduction in cell proliferation at 2.5 μM of FS within 6 h of treatment, whereas normal HEK-293 cells treated with FS showed least cytotoxicity, indicating that FS may be safe for normal cells (Figure 2B).

Since FS exhibited significant inhibition in cell proliferation in A549 cells, we were interested to know whether the observed reduction in cell numbers upon treatment was due to any arrest in the cell cycle. As anticipated, the treatment brought a significant arrest in the S and G2/M phases of the cell cycle with a concentration-dependent increase in the subG0 population (Figures 2C,D). In the cell cycle analysis, cells in the subG0 phase indicate an apoptotic population. The vehicle control (0.01% dimethyl sulfoxide) exhibited the characteristic cell cycle distribution pattern (Figure 2B), with G1 phase having 70 ± 1.3% cells, S phase having 9.5 ± 1.8% cells, G2/M phase having 11.5 ± 0.73% cells, and a sub-G0 phase corresponding to <5 ± 0.97%. The treatment, on other hand, showed a considerable decrease in the G1 population, with a significant increase in the proportion of cells in the subG0, S, and G2/M phases. After 24 h of treatment with 0, 2.5, 5, and 10 μM of FS, a dose-dependent increase in the subG0 cells was observed in A549 cells as compared with that of the untreated cells, with a high percentage found at a dose of 10 μM. Thus, FS induces apoptosis through arrest at the S and G2//M phases of the cell cycle.

**Figure 3:**
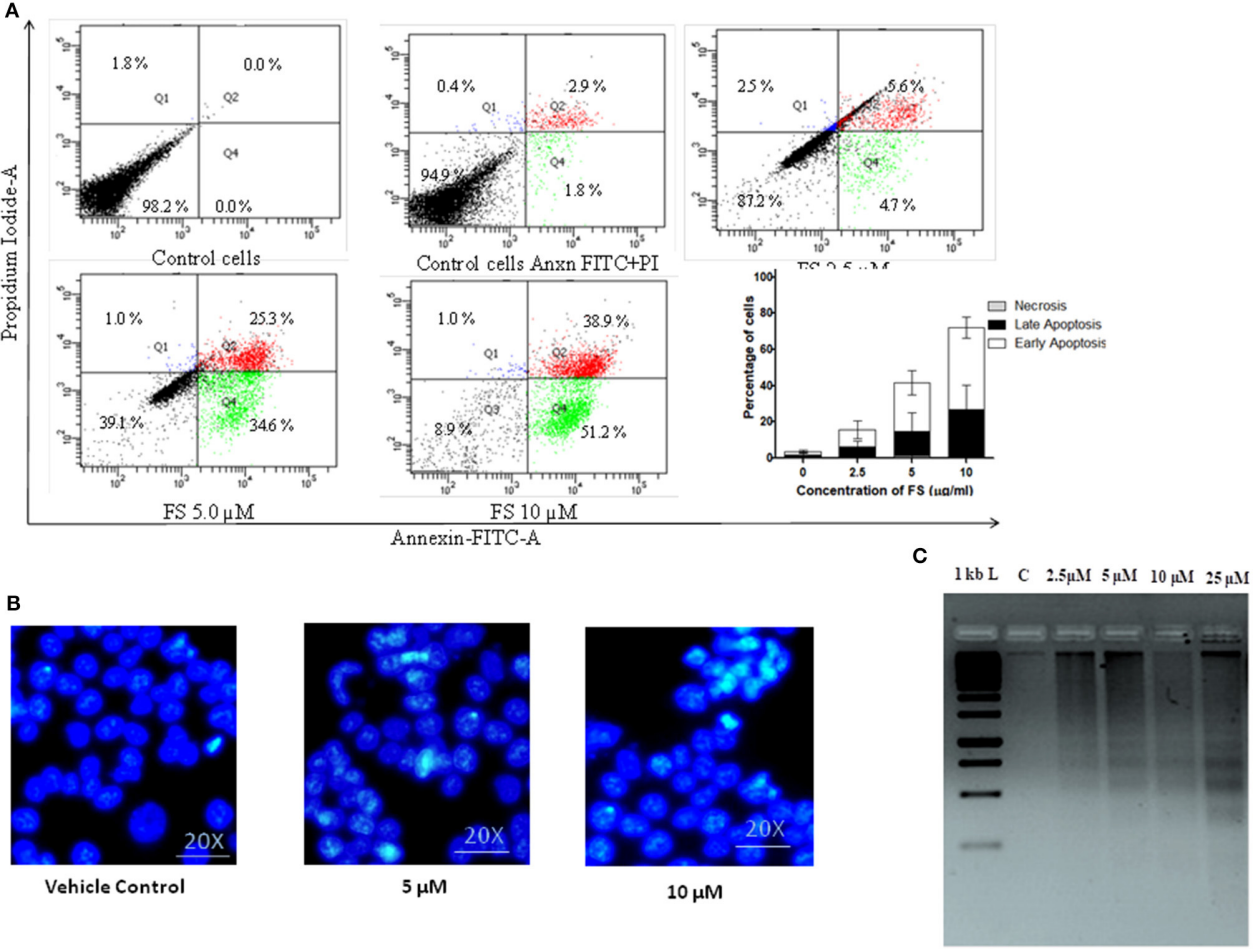
Fungal taxol (FS)-induced apoptosis in A549 cells. **(A)** Effect of FS treatment on phosphatidyl serine on plasma membrane externalization. The cells were treated with an indicative concentration of FS for 24 h, stained with annexin V–fluorescein isothiocyanate and propidium iodide (PI), and analyzed by flow cytometry. The live cells are both annexin V-negative and PI-negative (Q3—left lower quadrant), while the cells that are in early apoptosis are annexin V-positive and PI-negative (Q4—right lower quadrant), and the cells that are in late apoptosis or already dead are both annexin V-positive and PI-positive (Q2—right upper quadrant). The bar diagram represents the percentage of cells in the early and the late phases of apoptosis, respectively. Data are the means ± SD of three independent experiments. **(B)** Morphological changes of FS-treated A549 cells stained by DAPI. The cells were subjected to a 12-h treatment with FS. The occurrence of nuclear shrinkage was accompanied by chromatin condensation (indicated by arrows) in the cells. DMSO-treated (0.1%) cells served as vehicle control. **(C)** Effect of FS on DNA fragmentation assay in A549 cells. The cells were treated with indicated concentrations of FS for 24 h. M, DNA molecular size marker. DNA was extracted, and fragmentation was determined by agarose gel electrophoresis. The gel stained with ethidium bromide was viewed under a UV transilluminator.

Apoptosis is a dynamic cellular death process stimulated by normal physiological or pathological factors for the elimination of unwanted or damaged cells. Apoptotic cells have distinct morphologic features, characterized by loss of plasma membrane asymmetry and attachment, plasma membrane blebbing, condensation of the cytoplasm and nucleus, and DNA fragmentation or cleavage (29). The loss of plasma membrane asymmetry, the earliest event in apoptosis, is specified by translocation of membrane phosphatidylserine (PS) from the inner side of the plasma membrane to the outer surface of the cell. The loss of plasma membrane asymmetry in A549 cells treated with different concentrations of FS was confirmed using FITC-conjugated annexin V. Annexin V, a Ca^2+^-dependent phospholipid-binding protein, has high affinity for phosphatidyl serine. Because annexin V also binds to necrotic cells, the cells were counter-stained with PI to distinguish between apoptotic and necrotic cells. Viable cells with intact membranes exclude PI, whereas the membranes of dead and damaged cells are permeable to PI. The flow cytometry generated data were analyzed with FACS Diva 5 software. As represented in the plot (Figure 3A), the live cells are both annexin V-negative and PI-negative (Q3—left lower quadrant), while cells that are in early apoptosis are annexin V-positive and PI-negative (Q4—right lower quadrant), and cells that are in late apoptosis or already dead are both annexin V-positive and PI-positive (Q2—right upper quadrant). The results show that A549 cells treated with a low concentration (2.5 μM) of FS underwent apoptosis, with 4.7% in early apoptosis phase and 5.6% of cells in late apoptosis, and at an increasing concentration to 10 μM, it was observed that 51.2% cells were in early apoptosis and 38.9% of the cells were in late apoptosis phase (Figure 3A). These results suggest that FS may induce apoptosis in a concentration-dependent manner in A549 cells.

The other molecular events of apoptosis, like nuclear shrinkage and chromatin condensation, were further confirmed by nuclear staining with DAPI and DNA fragmentation assay (Figures 3B,C). As can be seen in Figure 3C, the treated cells exhibited nuclear shrinkage and chromatin condensation, the hallmark morphological changes of apoptosis. It has been proposed that DNA fragmentation is carried out by caspase-activated DNase (CAD). The activation of CAD leads to cleavage of nuclear DNA into multiples of ~200-bp oligonucleosomal-sized fragments (30). These characteristic patterns of DNA ladders are widely used as biochemical markers of apoptosis. There was a dose-dependent increase in DNA fragmentation pattern upon FS treatment as resolved by agarose gel (Figure 3B). These observations provide further evidence on nuclear dysfunction induced by FS in A59 cells. Thus, the detection of externalized phosphatidyl serine at the plasma membrane, nuclear condensation, and DNA fragmentation pattern confirmed that FS exhibits strong apoptosis-inducing potential (Figure 3).

### FS-Induced Apoptosis in A549 Cells Through Elevated ROS Production Mediated Through Intrinsic and Extrinsic Pathways

**Figure 4:**
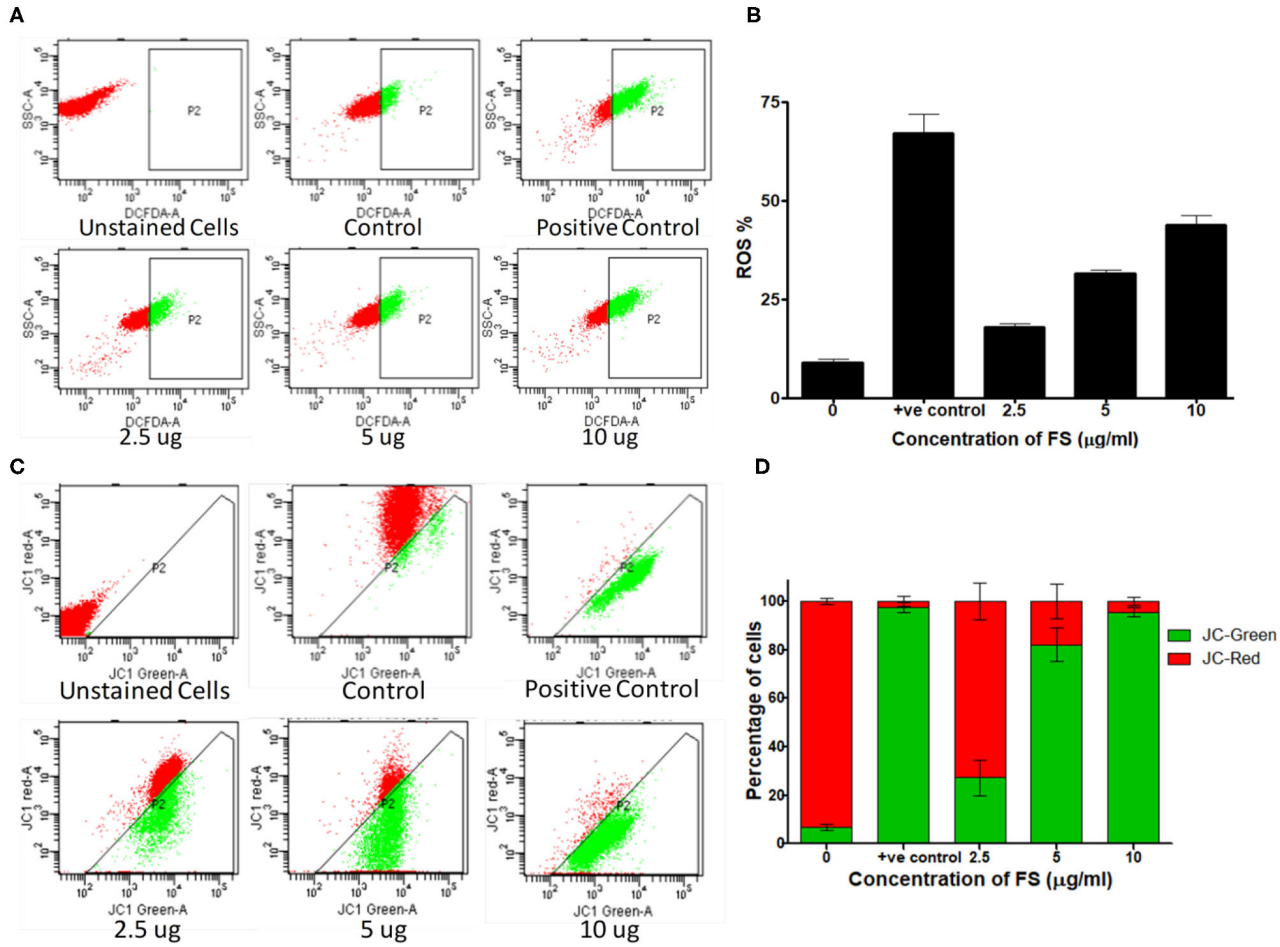
Mechanism of apoptosis induced by fungal taxol (FS). **(A)** Effect of FS on reactive oxygen species (ROS) production. Cells treated with the indicated concentration of FS were stained with 2′,7′-dichlorodihydrofluorescein diacetate dye, and the intracellular ROS was monitored by flow cytometry. **(B)** The bar diagram represents the percentage of cells that produced ROS. Data are presented as mean ± SEM, *n* = 3. **(C)** Effect of FS on the loss of mitochondrial membrane potential. Cells were treated with the indicated concentration of FS for 24 h and subsequently analyzed for changes in mitochondrial membrane potential by JC-1 staining using flow cytometry. **(D)** The bar diagram represents the percentage of JC-red and JC-green cells. Data are presented as mean ± SEM, *n* = 3.

Elevated intracellular levels of ROS cause damage to cellular macromolecules like proteins, nucleic acids, lipids, membranes, and organelles, which can lead to the activation of apoptosis. It is well established that chemotherapeutics exert their anticancer effects through the induction of oxidative stress and ROS-mediated apoptosis in cancer (31). To explore the role of ROS in the mechanism of apoptotic action of FS, we analyzed the effect of FS on intracellular ROS levels in A549 cells using the fluorescent dye DCFH-DA. The results (Figures 4A,B) show that FS enhanced the generation of ROS in a dose-dependent manner in the cells as compared to the untreated control. This indicates that the FS-induced apoptosis in A549 cells is mediated through the generation of ROS.

**Figure 5:**
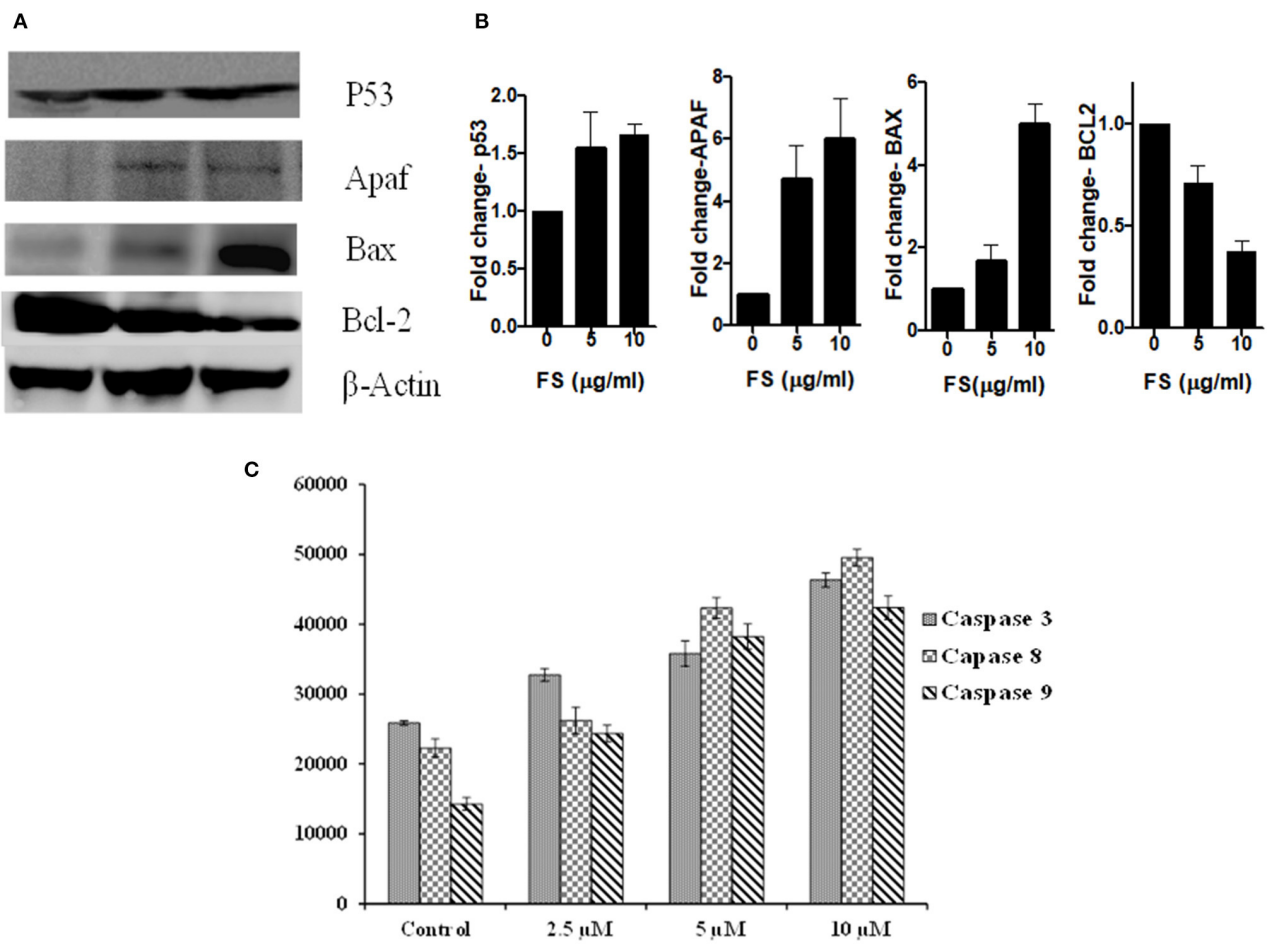
**(A)** Representative Western blots showing expression patterns of p53, Apaf, Bax, Bcl2, and β-Actin in FS treated A549 cells. **(B)** Bar diagram represent the relative protein quantification of Apaf-1, Bax, Bcl2, and p53, normalized to beta-actin. Data are presented as the mean ± SEM, *n* = 3. **(C)** Caspase-3, caspase-8, and caspase-9 activities were evaluated using caspase fluorometric assay kits (promega). Caspase activities were carried out using a fluorogenic caspase substrate measured with a fluorimeter. Data are the means ± SD of three independent experiments.

ROS-induced apoptosis can be elicited through the mitochondria-mediated intrinsic pathway and the death-receptor-mediated extrinsic pathway (32, 33). ROS can activate BAX (BCL2 Associated X) and upregulate the tumor suppressor protein, P53 (34). Cell cycle arrest and apoptosis are the most prominent outcomes of p53 activation. p53 activation arrests cells at the G_2_/M phases (35). We have found a significant upregulation of P53 in the cells treated with FS taxol. Cell cycle arrest by p53 is mainly mediated by the transcriptional activation of p21/WAF1 (36, 37). Once activated, Bax and Bak oligomerize to form pores in the mitochondrial outer membrane, leading to changes in the mitochondrial membrane potential and structure and release of cytochrome *c* (38). Cytosolic cytochrome *c* forms a complex with Apaf-1 and procaspase-9, called the apoptosome, in the presence of dATP, resulting in the auto-activation of caspase-9, which in turn activates the executioner caspases, resulting in cell death (39). Interestingly, PS triggered the up-regulation of BAX, with down-regulation in the protein BCL2, and induced the overexpression of tumor suppressor protein, Apaf-1 (Figures 5B,C). In addition, the activities of one of the effector caspases, caspase-3, and two initiator caspases, caspase 8 and caspase 9, in the cell-free extracts of FS-treated A549 cells detected using fluorogenic specific caspase substrates were significantly higher in the FS-treated cells compared with those in the control cultures (Figure 5A). The activation of caspase-8 for the extrinsic pathway and caspase-9 for the intrinsic pathway suggested that FS induced apoptosis through both intrinsic and extrinsic pathways.

The loss of mitochondrial membrane potential in FS-treated A549 cells (24 h) was measured by using flow cytometry with JC-1 dye (Figures 4C,D). The JC-1 dye shows potential-dependent accumulation in the mitochondria of viable cells, indicated by a fluorescence emission shift from green (~529 nm) to red (~590 nm). As shown in Figures 4C,D, a dose-dependent decrease in the red fluorescent J-aggregates with an increase in green fluorescent J-monomers upon FS treatment implies a depolarization of mitochondrial membrane potential. A combination of the abovementioned results suggests that FS triggered the generation of ROS in A549 cells and elicited cell death by both extrinsic as well as mitochondria-mediated intrinsic pathway of apoptosis.

In conclusion, the findings from our present study demonstrate that taxol, isolated from entophytic fungus, has a significant inhibitory effect on cancer cell proliferation in A549. Furthermore, the sub-acute oral administration of FS up to 500 mg/kg for a period of 28 days appears to be safe in rats and may not cause severe treatment-related toxicity. However, further clinical investigations are needed to confirm its safety and effectiveness in humans.

## Data Availability Statement

The raw data supporting the conclusions of this article will be made available by the authors, without undue reservation.

## Ethics Statement

The animal study was reviewed and approved by Institutional Animal Ethics Committe, Indian Institute of Science, Bangalore−560012, India.

## Author Contributions

GK, BA, MD, and CJ contributed towards developing the content, including participation in the concept, design, analysis, conducting experiments, writing, and revision of the manuscript. MJ conducted few experiments. RG helped in sub-acute toxicity studies.

## Conflict of Interest

The authors declare that the research was conducted in the absence of any commercial or financial relationships that could be construed as a potential conflict of interest.
